# Supplementary material for: Statistical signature of subtle behavioral changes in large-scale assays
Source: PLoS Comput Biol. 2025 Apr 21;21(4):e1012990. doi: 10.1371/journal.pcbi.1012990 (PMC12121925; doi:10.1371/journal.pcbi.1012990)
Supplement: S1 Text — Supporting information on the models described in the Methods section (PDF) [file pcbi.1012990.s001.pdf]

Supporting information for "Statistical signature of subtle behavioral changes in large-scale assays"

Alexandre Blanc[1,2*], François Laurent[1,2,3], Alex Barbier–Chebbah[1,2], Hugues Van Assel[9], Benjamin T. Cocanougher[4,5,6], Benjamin M.W. Jones [4,5,6], Peter Hague[4,5,6], Marta Zlatic[4,5,6], Rayan Chikhi[7], Christian L. Vestergaard[1,2], Tihana Jovanic[8*‡], Jean-Baptiste Masson[1,2*‡], Chloé Barré[1,2*‡]

1 Institut Pasteur, Université Paris Cité, CNRS UMR 3751, Decision and Bayesian Computation, Paris, France
2 Epiméthée, INRIA, Paris, France
3 Institut Pasteur, Université Paris Cité, Bioinformatics and Biostatistics Hub, Paris, France
4 University of Cambridge, Department of Zoology, Cambridge, United Kingdom
5 MRC Laboratory of Molecular Biology, Neurobiology Division, Cambridge, United Kingdom
6 Janelia Research Campus, Howard Hughes Medical Institute, Ashburn, Virginia, United States
7 G5 Sequence Bioinformatics, Department of Computational Biology, Institut Pasteur, Paris, France
8 Institut des Neurosciences Paris-Saclay, Université Paris-Saclay, Centre National de la Recherche Scientifique, UMR 9197, Saclay, France
9 École Normale Supérieure de Lyon, UMPA, Lyon, France

‡These authors contributed equally.
* aleblanc@pasteur.fr, cbarre@pasteur.fr, jbmasson@pasteur.fr, tihana.jovanic@cnrs.fr

Latent space

New behaviors

We tested the capacity of the latent space to represent new actions beyond the six classical ones [1, 2]. We show two examples of possible new action categories in Fig A of S1 Text, namely C-shape and head-tail. In the former, the larva takes the shape of a C with variable time spent in that state. It is observed, for example, prior to rolling. The larva exhibits hunch-like motion in the latter with rapid head and tail retraction. It is observed, for example, following air flow puffs.

Exploring the Latent Space

Beyond the usual dictionary of larval actions and behavior, the latent representation can be explored without labels.

Clustering the latent space. While clustering is not necessary for our analysis approaches, discrete behavior description can be instrumental in describing larva dynamics.

We used the persistence-based clustering algorithm ToMATo [3]. The algorithm requires an estimate of the density at the data points and the pairwise distances matrix between them to perform the clustering. The clustering joins a mode-seeking phase

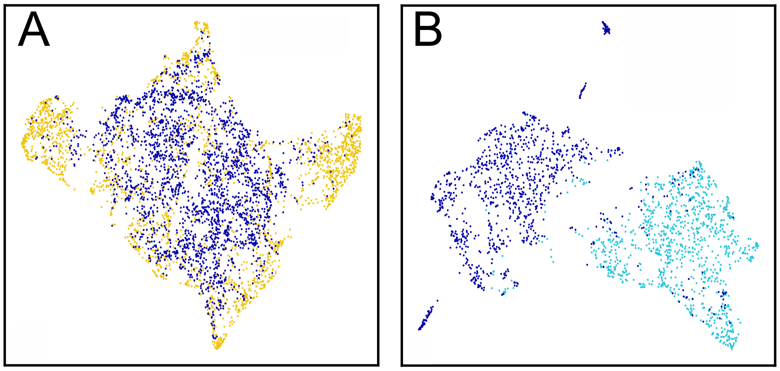

Fig A. Latent representations of C-shape (A)and Head-tail (B). (A) C-shapes: deep blue, rolls: yellow. (B) Head-tails: deep blue, backs: light blue.

based on a graph-based hill-climbing scheme and a topological persistence merging phase in the density map. Clustering was performed on the combined training and validation dataset.

In ToMATo [3], the number of clusters is controlled by a merging threshold, the minimum prominence a local peak must attain to be considered significant. A common practice to define the number of clusters is to use the gap statistic [4]. Instead of setting the number of clusters, we designed a graphical interface to examine the hierarchy dynamically. Interestingly, as in [5], one of the clusters identified through this procedure captures an anomaly in the larva tracking where the head and the tail are suddenly swapped.

Interface to navigate the latent space We developed a software tool which allows for interaction and visualization of the cluster hierarchy, a visualization of the 2D projection of the latent space, and generation of video data representing the samples in each cluster (Fig **??** of S1 Text).

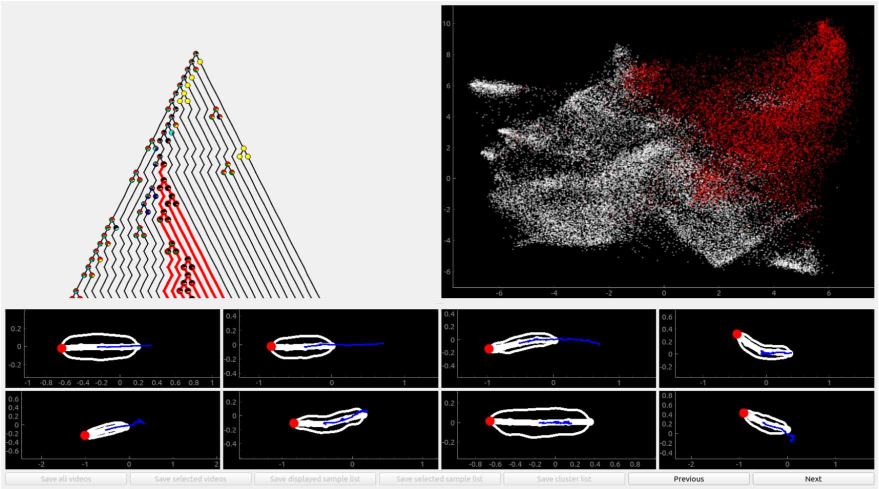

Fig B. Interface of the latent space navigation software: the clustering tree, the portion of the latent space selected, and examples of larva dynamical actions belonging to this cluster. Supplementary Videos 2. provides a video showing interaction with the software.

By interacting with the tree on the top left, we can choose a particular cluster to visualize. The cluster node and its children are then highlighted in the tree, while the corresponding data points are highlighted in the 2D projection on the top right. Samples from the cluster are displayed at the bottom of the interface. Various display settings can be used, such as the display of the outlines or the midlines of the recorded larva contours. The larva's head is highlighted in red, while the trace of its midpoint is plotted in blue. Finally, the depth of the cluster tree can be varied.

To declutter the tree view, the user can interactively fold a cluster, hiding all of its children from view, if they consider the distinctions between the different children clusters irrelevant. Note that these merges need not be consistent with the merging criterion of ToMATo, leaving the researcher with all the freedom to merge clusters, with the limitation that cluster merges must respect the tree structure.

Screen scale cluster definition

The screen can be used to define relevant cluster numbers. After computing the complete cluster hierarchy and associating each genotype to all clusters, we can prune the hierarchy to ensure that all clusters have at least one genotype belonging to them that is different from other clusters.

Generative model for sequences of discrete behaviors

The markovian generative model for sequences of discrete behaviors can be sampled using the following algorithm :

Algorithm 1 MCMC to generate behavioral sequences

```
T_end                                           ▷ Duration of simulation (in seconds)
K                                               ▷ Number of time step per second
i ← random.categorical(initial probabilities)   ▷ Initialize the larva's action
sequence ← [ ]
for s = 0 to T_end do

    for k = 0 to K do
        dt ← dt + k
        if random.uniform([0,1]) < (1 − e^{−λ_i(s)Δt}) then

            for n = 0 to 10 do
                append random.normal(μ_i(s, dt))) to features
            end for
            a ←random.choice(features)
            append (i, dt, a) to sequence
            i ←random.choice([0, . . . , N_behavior], weights = T_i(s))
            dt ← 0
            features ← [ ]
        end if
    end for
end for
return sequence
```

References

1. Jovanic T, Schneider-Mizell CM, Shao M, Masson JB, Denisov G, Fetter RD, et al. Competitive Disinhibition Mediates Behavioral Choice and Sequences in Drosophila. Cell. 2016;167(3):858–870.e19. doi:10.1016/j.cell.2016.09.009.

2. Masson JB, Laurent F, Cardona A, Barré C, Skatchkovsky N, Zlatic M, et al. Identifying Neural Substrates of Competitive Interactions and Sequence Transitions during Mechanosensory Responses in Drosophila. PLOS Genetics. 2020;16(2):e1008589. doi:10.1371/journal.pgen.1008589.

3. Chazal F, Guibas LJ, Oudot SY, Skraba P. Persistence-Based Clustering in Riemannian Manifolds. J ACM. 2013;60(6):41:1–41:38. doi:10.1145/2535927.

4. Tibshirani R, Walther G, Hastie T. Estimating the Number of Clusters in a Data Set via the Gap Statistic. Journal of the Royal Statistical Society: Series B (Statistical Methodology). 2001;63(2):411–423. doi:10.1111/1467-9868.00293.

5. Vogelstein JT, Park Y, Ohyama T, Kerr RA, Truman JW, Priebe CE, et al. Discovery of Brainwide Neural-Behavioral Maps via Multiscale Unsupervised Structure Learning. Science. 2014;344(6182):386–392. doi:10.1126/science.1250298.
