# Supplementary figures and images for: Statistical signature of subtle behavioral changes in large-scale assays
Source: PLoS Comput Biol. 2025 Apr 21;21(4):e1012990. doi: 10.1371/journal.pcbi.1012990 (PMC12121925; doi:10.1371/journal.pcbi.1012990)

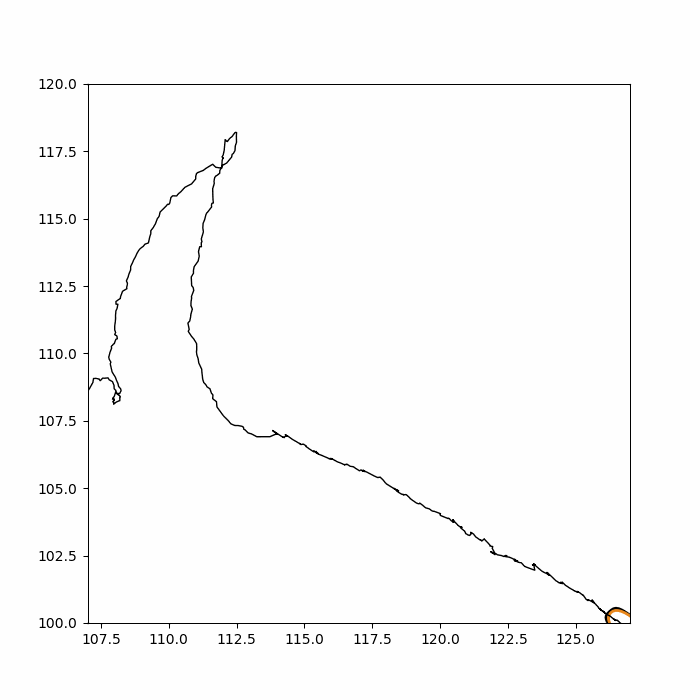

Supplement: S1 Video — Video version of Fig 2C. (GIF) [file pcbi.1012990.s002.gif]
