# Supplementary material for: Statistical signature of subtle behavioral changes in large-scale assays
Source: PLoS Comput Biol. 2025 Apr 21;21(4):e1012990. doi: 10.1371/journal.pcbi.1012990 (PMC12121925; doi:10.1371/journal.pcbi.1012990)
Supplement: Attachment — Submitted filename: rebuttal-Blanc.pdf [file pcbi.1012990.s012.pdf]

We thank the reviewer for his careful review of the paper and positive opinion. We addressed all his/her remarks.

Reviewer #1: *Blanc et al* extend prior work by Masson and colleagues in automating the quantitative analysis of Drosophila larval behavioral dynamics from video microscopy. The intelligent use of a simple physical model of larval shape -- not much more than the physics of an elastic larva-shaped object -- is a principled way to regularize the extraction of larval posture from video. Temporal continuity is incorporated into the analysis in a straightforward way using an autoencoder that is fed multiple successive time points. The system seems like it robustly assigns behavioral categories to every time point, and effectively assigns quantitatively-rich behavioral phenotypes spanning state probabilities and transition rates.

The field now needs toolboxes like this one. Large Drosophila libraries with well-defined genetic perturbations demand automated methods for quantitative phenotyping. As the field moves more deeply into higher-order behavioral decision-making, these phenotypes are likely to be more nuanced. Quantitatively significant but visibly subtle differences between animals may harbor important insights into the workings of brain circuits.

I have no major suggestions. The paper is highly technical, but generally reads well. There are *many* typos and misspellings. The entire paper needs careful proofreading. I didn't try to proofread the equations. I only skimmed the equations for general plausibility. The authors would do well to go through the entire manuscript with a fine-toothed comb.

The paper was carefully proofread. British spelling was changed to American spelling across the entire manuscript (behaviour→behavior, regularise→regularize, …) and every spelling mistake spotted was corrected. Where adequate, we corrected syntax, replacing use of the first person plural with the passive voice and reordering words where needed. Some sentences were split in shorter sentences for clarity. We also corrected grammatical mistakes such as describing figures as “at the top, at the bottom” instead of “on top, on the bottom”. Finally, we added a detailed list of author contributions.
